# Relationship of anabolic and catabolic biomarkers with muscle strength and physical performance in older adults: a population-based cross-sectional study

**DOI:** 10.1186/s12891-015-0654-7

**Published:** 2015-08-19

**Authors:** Yongxia Meng, Hongmei Wu, Yi Yang, Huanmin Du, Yang Xia, Xiaoyan Guo, Xing Liu, Chunlei Li, Kaijun Niu

**Affiliations:** Chinese People’s Liberation Army 254 Hospital, Tianjin, China; Nutritional Epidemiology Institute and School of Public Health, Tianjin Medical University, Tianjin, China; Tianjin Centers for Disease Control and Prevention, Tianjin, China

## Abstract

**Background:**

Previous studies have found inflammation, growth factors, and androgen signaling pathways all contribute to sarcopenia. However, few studies simultaneously have investigated the association between these potential risk factors and sarcopenia among older people. The aim of the study was to investigate whether elevated levels of inflammatory cytokines combined with low levels of anabolic hormone have a synergy effect on muscle strength and functional decline in older people.

**Methods:**

We designed a cross-sectional study of 1,131 subjects aged 60 years and older. Concentrations of serum C-reactive protein, insulin-like growth factor 1 and dehydroepiandrosteronesulphate were assessed using chemiluminescent immunoassays. Handgrip strength was measured using a dynamometer, and physical performance was assessed using a four-meter gait speed and Timed Up and Go test. We defined poor physical performance as a 4-m gait speed <0.8 m/s or Timed Up and Go test ≥13.5 s.

**Results:**

After adjustment for potential confounding factors, in multiple linear regression analysis, C-reactive protein levels are inversely related to handgrip strength (*P* <0.01), and in multiple logistic regression analysis, C-reactive protein levels are inversely related to poor physical performance (*P* for trend <0.05) in males, but not in females. After combining three biomarkers, no significant results were observed between biomarker scores and muscle strength or physical performance.

**Conclusions:**

In older males, higher serum C-reactive protein levels, but not insulin-like growth factor 1 and dehydroepiandrosteronesulphate levels, are independently related to lower muscle strength and poor physical performance. In this study we did not observe that a combination of higher catabolic biomarkers and lower anabolic biomarkers were better predictors for muscle strength and physical performance.

## Background

Sarcopenia is a syndrome characterized by progressive and generalized loss of skeletal muscle mass, muscle strength and function [1]. Sarcopenia is an important clinical problem that impacts millions of older adults, and can lead to a multitude of adverse consequences, such as frailty, disability, morbidity, mortality, and higher fall risk [2–4].

Previous studies have found inflammation, growth factors, and androgen signaling pathways all contribute to sarcopenia. An extensive literature has shown that higher levels of inflammatory markers [5–9] and low levels of anabolic hormones were respectively associated with muscle strength and physical performance decline in older people [10–12]. Maintenance of muscle mass depends on the balance between protein synthesis and degradation, which are mediated by anabolic and catabolic signaling pathways [13, 14]. For example, the insulin-like growth factor 1 (IGF-1) pathway actives protein synthesis and inhibits degradation, thereby controlling the balance of muscle protein turnover; thus, a decline in hormones including IGF-1 and dehydroepiandrosteronesulphate (DHEAS), may contribute to development of sarcopenia [15–18]. Moreover, since serum levels of proinflammatory cytokines including interleukin-6 (IL-6) and tumor necrosis factor-alpha increase with age, proinflammatory pathway activation could contribute to muscle degradation and decreased protein synthesis [19]. Based on above, inflammation, growth factors, and androgen signaling pathways all contribute to sarcopenia. However, to date, few studies simultaneously have investigated the association between these potential risk factors and sarcopenia among older people. Whether elevated levels of inflammatory cytokines combined with low levels of anabolic hormone have a synergy effect on muscle strength and physical performance decline in older persons has not been explored. Therefore, we performed a cross-sectional study to explore the effect of high levels of catabolic biomarkers combined with low levels of anabolic biomarkers on muscle strength and physical performance in Chinese older adults.

## Methods

### Study participants

Our study population comprised of subjects aged 60 years and older living in the Hangu area of Tianjin City, one of the major cities of China. Face-to-face questionnaires were carried out by specially-trained interviewers.

Exclusion criteria were aimed to minimize confounding effects on sarcopenia. The following subjects were excluded: (1) who were younger than 60 years (*n* = 24); (2) whose serum DHEAS concentrations had not been measured (*n* = 112); (3) whose serum C-reactive protein (CRP) concentrations had not been measured (*n* = 4); (4) Subjects whose hand was injured and subjects who cannot walk by themselves or by the assistance of others or instruments were excluded (*n* = 8). In addition, there were 193 subjects who have not measured the 4-m gait speed. As a result of these exclusions, the final cross-sectional study population for muscle strength and TUGT analysis were composed of 1,131 subjects [age 68.95 ± 6.94 years (mean ± standard deviation, SD); males: 47.39 %], and for 4-m gait speed analysis was composed of 938 subjects. The protocol used here was approved by the Institutional Review Board of Tianjin Medical University, and participants provided informed consent for analysis of their data.

### Measurement of blood samples

Overnight fasting blood samples were collected the next morning. Serum IGF-1, CRP, DHEAS levels were determined using chemiluminescent immunoassay kits (Snibe, Shenzhen, China) according to the manufacturer instructions. The detection limit of this assay is 0.13 ng/ml for CRP, 5.0 ng/ml for IGF-1, and 1 μg/dl for DHEAS. Intra-assay coefficient of variation for CRP, IGF-1 and DHEAS were 7.8, 6.4 and 8.2 % respectively, and inter-assay coefficient of variation for CRP, IGF-1 and DHEAS were 10.2, 12.1 and 11.3 % respectively.

### Muscle strength

Evidence indicates that handgrip strength is highly correlated with mortality in older or pathological persons [20, 21]. Therefore, handgrip strength was chosen as an indicator of overall muscle strength [22]. Handgrip strength was measured using a dynamometer (EH101; CAMRY, Guangdong, China). Dynamometer width was adjusted for optimal fit for each participant. Participants were instructed to stand upright and with the dynamometer beside but not against their body. Participants were asked perform 2 maximum force trials for each hand and the measurements were recorded in kilograms. The maximum value attained during the four trials was used as the final score.

### Physical performance

Four-meter gait speed and Timed Up and Go test (TUGT) are a widely used criterion in geriatric assessment; we used these measurements in order to assess physical performance [1]. Four-meter gait speed has been shown to be associated with survival in older adults [23], and has also been shown to reflect health and functional status [24]. It has thus been recommended as a potentially useful clinical indicator of wellbeing among older adults [25].

#### TUGT

The subjects were seated in a free-standing padded armchair and stand up without use of arms, walk at a comfortable and safe pace to a line on the floor three meters away, turn and walk back to the chair and sit down again [26]. Time was measured while they rise from an arm chair until complete the series of functionally task.

The TUGT is recommended as a routine screening test for falls in guidelines published by the American Geriatric Society and the British Geriatric Society [27], a score of ≥13.5 s is used as a cut-point to identify those at increased risk of falls in the community setting [28]. Thus we considered a score of ≥13.5 s indicated a low physical performance.

#### Four-meter gait speed

Gait speed is considered a simple indicator of health status and of survival in older persons [29]. It can be used as a quick, safe, inexpensive and highly reliable instrument implemented to assess the physical function [30]. Participants walk for a 4 m distance at usual pace from a standing start [23]. Each one was allowed walk two trials, and the average speed was used for analyses. A walking speed at the cut-off point of 0.8 m/s has been recommended as measurement of sarcopenia [1].

### Assessment of other variables

We use a standardized, structured interview questionnaire to gather information about lifestyle factors and comorbidities. Physical activity (PA) in the most recent week was assessed using the short form of the International Physical Activity Questionnaire (IPAQ). Metabolic equivalent (MET) hours per week were calculated using corresponding MET coefficients (3.3, 4.0 and 8.0, respectively) according to the following formula [31]: MET coefficient of activity × duration (hours) × frequency (days). Total physical activity levels were assessed by combining separate scores for different activities.

Anthropometric parameters (height and body weight) were recorded for each participant using a standardized protocol. Body mass index (BMI) was calculated as weight (kg)/height (m^2^).

### Statistical analysis

Study population characteristics according to sex are reported as means (standard deviation) or median (interquartile range) values for continuous variables and proportions for categorical variables. Pearson’s simple correlation coefficients (*r*) were calculated to evaluate the correlation between log-transformed biomarker (CRP, IGF-1 or DHEAS) concentration and age. Correlation of CRP, DHEAS, and IGF-1 with one another was also determined by simple Pearson’s correlation. To explore the association between handgrip strength and serum biomarkers, multiple linear regression analysis and analysis of covariance (ANCOVA) were used simultaneously. The value of handgrip strength was used as the dependent variable, and the serum biomarker concentration levels were used as the independent variables. Multiple logistic regression analysis was used to examine the relationships between the tertiles of biomarkers (CRP, IGF-1 and DHEAS) and poor physical performance (TUGT ≥13.5 s or 4-m gait speed <0.8 m/s) after adjustment for covariates. Odds ratio (OR) and 95 % confidence interval (CI) were calculated. In order to investigated whether anabolic and catabolic biomarkers impact handgrip strength and physical performance simultaneously, three serum biomarkers were divided into tertiles: the lowest tertile of IGF-1 and DHEAS recorded as 1, the middle tertile of IGF-1 and DHEAS recorded as 2, and the highest tertile of IGF-1 and DHEAS recorded as 3; and the low tertile of CRP recorded as 3, the middle tertile of CRP recorded as 2, the high tertile of CRP recorded as 1. Then, we created variable biomarker scores (ranging from 3 to 9) that were computed as follows: biomarker scores = CRP score + IGF-1 score + DHEAS score. Then, we examined the relationship between the biomarker scores categories and handgrip strength or poor physical performance.

All statistical tests were two-tailed and a significant difference was defined as *P* <0.05. All statistical analyses were performed using Statistical Analysis System version 9.3 (SAS Institute Inc., Cary, NC, USA).

## Results

**Fig. 1:**
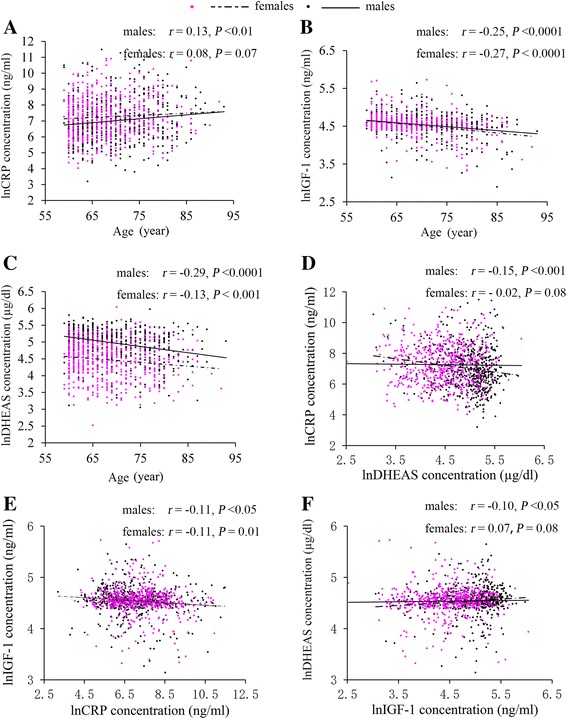
**a-c**. The Pearson’s simple correlation between biomarkers (C-reactive protein, insulin-like growth factor 1 and dehydroepiandrosteronesulphate) and age according to gender; **d-f**. The Pearson’s simple correlation of three biomarkers (C-reactive protein, insulin-like growth factor 1 and dehydroepiandrosteronesulphate) between each other

The study cohort included 1,131 subjects, with a mean ± SD age of 69.0 ± 6.9. Of these, 47.4 % were males (Table 1). Log-transformed CRP concentration increased with age in males (*r* = 0.13, *P* <0.01), and although not statistically significant, log-transformed CRP also correlated positively to age in females (*r* = 0.08, *P* = 0.07). IGF-1 concentration decreased with age both in males (*r* = −0.25, *P* <0.0001) and in females (*r* = −0.27, *P* <0.0001). Likewise, DHEAS concentration also decreased with age in both sexes (*r* = −0.29, *P* <0.0001 in males; *r* = −0.13, *P* <0.001 in females). In males, CRP, DHEAS, IGF-1 significantly correlated with one another (CRP and DHEAS: *r* = −0.15, *P* <0.001; CRP and IGF-1: *r* = −0.11, *P* <0.05; DHEAS and IGF-1: *r* = 0.10, *P* <0.05); however, in females, only CRP and IGF-1 displayed a significant correlation (*r* = −0.11, *P* <0.01) (Fig. 1).

**Table 1:** The characteristics of the study population^a^

	All	Males	Females
Characteristic	(*n* = 1131)	(*n* = 536)	(*n* = 595)
Age, years	69.0 (6.94) ^b^	69.9 (7.18)	68.1 (6.62)
BMI, kg/m^2^	25.4 (3.57)	25.1 (3.09)	25.7 (3.94)
Grip, kg	26.5 (9.37)	32.4 (7.94)	20.4 (5.43)
TUGT, s	11.2 (4.67)	11.0 (4.75)	11.4 (4.60)
4-m gait speed, m/s	0.99 (0.21) (*n* = 938)	1.03 (0.22) (*n* = 428)	0.95 (0.19) (*n* = 510)
CRP, mg/L	1.11 (0.49, 3.00) ^c^	0.93 (0.43, 2.50)	1.36 (0.55, 3.48)
IGF-1, ng/mL	97.5 (86.5, 105.9)	95.1 (86.7, 107.1)	93.8 (86.3, 104.6)
DHEAS, ug/dL	127.7 (77.6, 175.0)	162.7 (115.8, 197.1)	91.7 (61.0, 129.4)
Smoking, %			
Current	32.1	39.2	25.6
Former	19.6	27.7	12.4
Never	48.3	33.1	62.0
Drinking, %			
Current	28.3	52.4	6.50
Fomer	11.8	22.7	1.88
Never	59.9	24.9	91.6
PA, METs-h/week	23.1 (3.93, 76.7)	30.8 (1.40, 88.6)	23.1 (4.95, 69.3)
Depression symptoms, %	14.5	13.9	15.0
Fall, %	20.7	17.3	23.7
Hypertension, %	37.0	29.5	43.7
Diabetes, %	14.2	11.4	16.6
Cancer, %	1.95	1.30	2.52
CVD, %	20.7	14.0	26.7
Anemia, %	1.24	0.75	1.68

^a^ *BMI* body mass index, *TUGT* timed up and go test, *CRP* C-reactive protein, *IGF-1* Insulin-like growth factor-1, *DHEAS* dehydroepiandrosteronesulphate, *PA* physical activity, *CVD* cardiovascular disease

^b^Values are expressed as mean (standard deviation) (all such values)

^c^Values are expressed as median (interquartile range) (all such values)

Multiple linear regression analyses were performed to confirm the relationship between biomarkers concentration and handgrip strength. Log-transformed CRP, IGF-1, DHEAS levels correlated significantly with muscle strength in males using model 1 and model 2 (Table 2). In model 3, muscle strength distinctly showed a significant relationship with log-transformed CRP [standard regression coefficient (SRC) = −0.11; *P* <0.01] in males but not in females (SRC = −0.01; *P* = 0.76) after additionally adjusting for age. There was no significant relationship between log-transformed IGF-1 and muscle strength in either males or females (SRC = 0.04; *P* = 0.30 and SRC = 0.00; *P* = 0.80 respectively) after adjusting for potential confounders. Likewise, log-transformed DHEAS values were not a significant determinant of muscle strength (SRC = 0.05; *P* = 0.17 and SRC = 0.01; *P* = 0.71 in males and females respectively).

**Table 2:** The relationship between biomarkers and handgrip strength: multiple linear regression analysis^a^

	Handgrip strength					
	Males			Females		
	*β*	standard *β*	*P*	*β*	standard *β*	*P*
Model 1^b^						
CRP	−0.99	−0.17	<0.001	−0.20	−0.05	0.27
IGF-1	4.87	0.18	<0.001	2.44	0.14	<0.001
DHEAS	3.14	0.19	<0.001	0.90	0.09	<0.05
Model 2^c^						
CRP	−0.90	−0.15	<0.001	−0.20	−0.04	0.26
IGF-1	3.48	0.13	<0.01	2.01	0.11	<0.01
DHEAS	2.94	0.17	<0.001	0.77	0.08	0.06
Model 3^d^						
CRP	−0.61	−0.11	<0.01	−0.04	−0.01	0.76
IGF-1	1.08	0.04	0.30	−0.17	0.00	0.80
DHEAS	0.90	0.05	0.17	0.13	0.01	0.71

^a^ *CRP* C-reactive protein, *IGF-1* Insulin-like growth factor-1, *DHEAS* dehydroepiandrosteronesulphate

^b^Crude

^c^Adjusted for body mass index, smoking status, drinking status, physical activity and comorbidities of (hypertension, diabetes, cancer, cardiovascular disease, and anemia)

^d^Additionally adjusted for age

Table 3. shows the crude and adjusted relationship between physical performance (TUGT and 4-m gait speed) in males and females participants. In the final multivariate models, the adjusted OR (95 % CI) for TUGT across CRP tertiles were 1.00 (reference), 1.09 (0.5, 2.39), and 2.7 (1.34, 5.66) (*P* for trend <0.01) in males. The adjusted OR (95 % CI) for 4-m gait speed across CRP tertiles were 1.00 (reference), 1.25 (0.55, 2.88), and 2.84 (1.33, 6.33) (*P* for trend <0.05) in males. However, significant results of TUGT and 4-m gait speed across CRP tertiles were not observed in females. The reason of the association between CRP and muscle strength and physical performance was sex-specific is not clear. Gender difference may be related to the tendency of healthier life styles (eg. the percentage of women smokers and current drinkers is lower in women than in men) in females than males. Significant results of TUGT and 4-m gait speed across IGF-1 and DHEAS tertiles were not observed both in males and females.

**Table 3:** Adjusted relationships of tertiles of biomarkers to the TUGT and 4-m gait speed^a^

	Tertiles of biomarkers (Males)				Tertiles of biomarkers (Females)			
	Low	Middle	High		Low	Middle	High	
Tertiles of CRP (range: mg/L)	0.02-0.31 (*n* =178)	0.31-0.93 (*n* = 179)	0.93-3.86 (*n* = 179)	*p* for trend ^b^	0.01-0.42 (*n* = 198)	0.42-1.36 (*n* = 198)	1.36-4.74 (*n* = 199)	*p* for trend
No. of TUGT ≥ 13.5 s	15	16	39	-	28	29	35	-
Crude	Ref	1.15 (0.55, 2.40) ^c^	2.97 (1.6, 5.78)	<0.001	Ref	0.97 (0.55, 1.70)	1.26 (0.74, 2.17)	0.59
Model 1	Ref	1.09 (0.51, 2.36)	2.58 (1.32, 5.23)	<0.01	Ref	0.63 (0.32, 1.20)	0.92 (0.49, 1.71)	0.41
Model 2	Ref	1.11 (0.51, 2.44)	2.81 (1.41, 5.85)	<0.01	Ref	0.61 (0.31, 1.17)	0.91 (0.49, 1.71)	0.36
Model 3	Ref	1.09 (0.50, 2.39)	2.7 (1.34, 5.66)	<0.01	Ref	0.58 (0.29, 1.12)	0.86 (0.46, 1.62)	0.33
Tertiles of IGF-1 (range: ng/mL)	18.1-81.8 (*n* =178)	81.8-95.1 (*n* = 179)	95.1-112.9 (*n* = 179)		5.87-82.5 (*n* =198)	82.5-93.8 (*n* = 196)	93.8-109.9 (*n* = 201)	
No. of TUGT ≥ 13.5 s	26	20	24	-	41	19	32	-
Crude	Ref	0.67 (0.35, 1.25)	0.88 (0.48, 1.59)	0.62	Ref	0.43 (0.24, 0.76)	0.72 (0.43, 1.19)	0.01
Model 1	Ref	0.86 (0.44, 1.66)	1.24 (0.65, 2.38)	0.62	Ref	0.65 (0.33, 1.23)	1.19 (0.64, 2.2)	0.14
Model 2	Ref	0.84 (0.42, 1.66)	1.29 (0.66, 2.53)	0.53	Ref	0.61 (0.31, 1.18)	1.19 (0.65, 2.22)	0.10
Model 3	Ref	0.85 (0.42, 1.72)	1.27 (0.65, 2.49)	0.60	Ref	0.60 (0.30, 1.17)	1.12 (0.60, 2.11)	0.14
Tertiles of DHEAS (range: ug/dL)	21.2-0.31 (*n* =178)	0.31-0.93 (*n* = 179)	0.93-3.86 (*n* = 179)		12.4-50.1 (*n* = 198)	50.1-91.7 (*n* = 198)	91.7-150.8 (*n* = 199)	
No. of TUGT ≥ 13.5 s	34	20	16	-	39	22	31	-
Crude	Ref	0.54 (0.29, 0.97)	0.42 (0.22, 0.78)	<0.05	Ref	0.54 (0.31, 0.94)	0.73 (0.43, 1.23)	0.06
Model 1	Ref	0.89 (0.46, 1.7)	0.75 (0.37, 1.48)	0.68	Ref	0.65 (0.34, 1.2)	1.09 (0.6, 1.98)	0.15
Model 2	Ref	0.88 (0.45, 1.69)	0.73 (0.36, 1.47)	0.64	Ref	0.64 (0.34, 1.2)	1.06 (0.58, 1.95)	0.15
Model 3	Ref	0.93 (0.47, 1.82)	0.73 (0.35, 1.47)	0.64	Ref	0.71 (0.37, 1.33)	1.09 (0.59, 2.01)	0.25
Tertiles of CRP (range: mg/L)	0.02-0.31 (*n* =146)	0.31-0.93 (*n* = 147)	0.93-3.86 (*n* = 135)		0.01-0.42 (*n* = 181)	0.42-1.36 (*n* = 165)	1.36-4.74 (*n* = 164)	
No. of 4 m gait speed < 0.8 m/s	14	15	29	-	30	31	27	-
Crude	Ref	1.09 (0.50, 2.37)	2.6 (1.33, 5.30)	<0.01	Ref	1.08 (0.62, 1.89)	0.97 (0.55, 1.7)	0.82
Model 1	Ref	1.13 (0.51, 2.55)	2.43 (1.18, 5.19)	<0.05	Ref	0.76 (0.40, 1.43)	0.77 (0.41, 1.44)	0.72
Model 2	Ref	1.26 (0.56, 2.89)	2.88 (1.36, 6.37)	<0.05	Ref	0.74 (0.39, 1.39)	0.75 (0.4, 1.4)	0.65
Model 3	Ref	1.25 (0.55, 2.88)	2.84 (1.33, 6.33)	<0.05	Ref	0.72 (0.37, 1.37)	0.72 (0.37, 1.37)	0.59
Tertiles of IGF-1 (range: ng/mL)	18.1-81.8 (*n* =124)	81.8-95.1 (*n* = 159)	95.1-112.9 (*n* = 145)		5.87-82.5 (*n* =153)	82.5-93.8 (*n* = 175)	93.8-109.9 (*n* = 182)	
No. of 4 m gait speed < 0.8 m/s	19	18	21	-	29	22	37	-
Crude	Ref	0.63 (0.31, 1.26)	0.9 (0.46, 1.75)	0.57	Ref	0.68 (0.37, 1.22)	1.05 (0.61, 1.82)	0.13
Model 1	Ref	0.73 (0.35, 1.52)	1.2 (0.59, 2.47)	0.49	Ref	0.88 (0.46, 1.70)	1.31 (0.70, 2.47)	0.24
Model 2	Ref	0.73 (0.34, 1.54)	1.23 (0.59, 2.59)	0.46	Ref	0.94 (0.48, 1.82)	1.36 (0.72, 2.6)	0.26
Model 3	Ref	0.73 (0.34, 1.55)	1.21 (0.58, 2.56)	0.48	Ref	0.92 (0.46, 1.82)	1.14 (0.59, 2.23)	0.59
Tertiles of DHEAS (range: ug/dL)	21.2-0.31 (*n* =130)	0.31-0.93 (*n* = 142)	0.93-3.86 (*n* = 156)		12.4-50.1 (*n* = 166)	50.1-91.7 (*n* = 169)	91.7-150.8 (*n* = 175)	
No. of 4 m gait speed < 0.8 m/s	24	19	15	-	39	21	28	-
Crude	Ref	0.68 (0.35, 1.30)	0.47 (0.23, 0.94)	0.10	Ref	0.49 (0.27, 0.87)	0.6 (0.35, 1.04)	<0.05
Model 1	Ref	0.98 (0.49, 1.98)	0.72 (0.34, 1.49)	0.62	Ref	0.64 (0.34, 1.19)	0.86 (0.47, 1.57)	0.26
Model 2	Ref	0.95 (0.46, 1.95)	0.7 (0.32, 1.47)	0.60	Ref	0.62 (0.33, 1.16)	0.81 (0.44, 1.49)	0.23
Model 3	Ref	1.01 (0.48, 2.09)	0.68 (0.31, 1.44)	0.52	Ref	0.74 (0.38, 1.41)	0.86 (0.46, 1.62)	0.52

Model 1: adjusted for age and BMI

Model 2: adjusted for age, BMI, smoking status, and drinking status

Model 3: adjusted for age, BMI, smoking status, drinking status, physical activity and family history of cardiovascular disease, hypertension, diabetes, and cancer

^a^ *BMI* body mass index, *TUGT* timed up and go test, *CRP* C-reactive protein, *IGF-1* Insulin-like growth factor-1, *DHEAS* dehydroepiandrosteronesulphate

^b^Analysis by multiple logistic regression analysis

^c^Values are expressed as odds ratios (95 % confidence interval) (all such values)

**Fig. 2:**
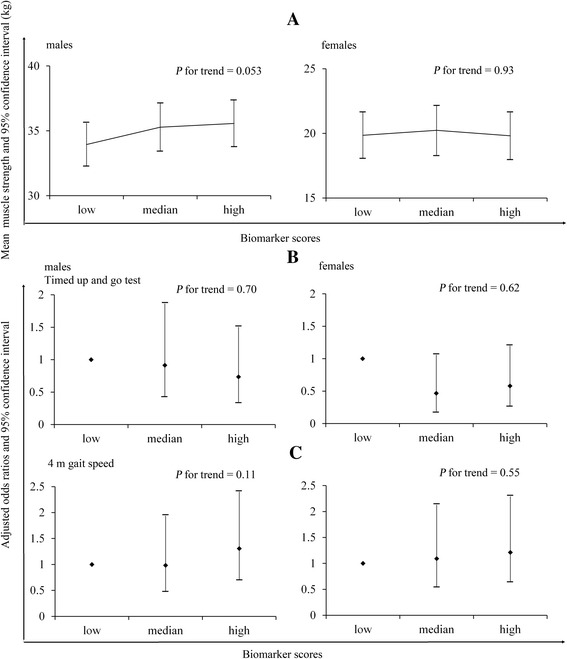
The relationship between biomarker scores and muscle strength and physical performance. **a** The adjusted relationship between biomarker scores and muscle strength. **b** The adjusted relationship between biomarker scores and TUGT. **c** The adjusted relationship between biomarker scores and 4-meter gait speed

Figure 2a. displays the relationship between biomarker scores and muscle strength. Covariance analysis (ANCOVA) showed that in the final multivariate models, significance values were attenuated and were not statistically significant (*P* for trend = 0.05 in males, *P* for trend = 0.93 in females). This suggests that the association between muscle strength and serum biomarkers score is likely acting through an aging pathway.

Figure 2b and cdisplay the adjusted relationship between biomarker scores and TUGT/4-m gait speed. However, significant results of TUGT and 4-m gait speed across biomarker scores were not observed both in males and females.

## Discussion

This cross-sectional study was conducted to assess the association between serum biomarker levels (CRP, IGF-1, and DHEAS) and muscle strength, physical performance (TUGT and 4-m gait speed) in older individuals. We find that elevated serum CRP is independently related to muscle strength and physical performance in males. In this study, we did not observe that a combination of higher catabolic biomarkers and lower anabolic biomarkers were better predictors for muscle strength and physical performance.

These findings partially agree with several previous studies that showed a significant association between high C-reactive protein levels and the decline of physical activity [32] or muscle strength [33] in older adults. In our study, significant relationship between high CRP levels and lower muscle strength and physical performance remained even when we adjusted for a number of potential confounders. Whether or not CRP directly affects skeletal muscle function is unknown; however, results of experimental studies [34] support a direct link between inflammatory markers and muscle mass. The inverse relationship between serum CRP levels and muscle strength and physical performance demonstrated in this study is most likely explained by the catabolic effect of inflammatory markers on muscle tissue. Increased circulating levels of cytokines are associated with a progressive increase in glucocorticoid and catecholamine levels and suppression of the IGF1-Akt [also known as protein kinase B (PKB)] pathway [35]. Nuclear factor-κB (NFκB) transcription factors, which play major roles as mediators of immunity and inflammation, are expressed in skeletal muscle, where they mediate the effect of inflammatory cytokines on muscle wasting and cachexia. Pro-inflammatory cytokines are potent stimulants of proteolysis through the NFκB signaling pathway [36].

Aging is associated with a variable decline of several hormones, especially sex hormones (*e.g*. testosterone and DHEAS) and growth hormones (*e.g*. growth hormone and IGF-1) [37]. IGF-1 is a potent anabolic hormone mediating muscle growth and regeneration [38]. Experimental studies have shown that systemic IGF-1 administration increases the rate of skeletal muscle functional recovery after injury [39]. DHEAS is a major androgen in circulation that is secreted by adrenal cortex [40]. The fact that DHEAS levels decrease with age, means that it likely plays an important role in age-related onset of sarcopenia. DHEAS may induce beneficial age-related effects on body composition and physical performance.

Although hormone status has an important relationship with strength in older adults, our study failed to find a positive relationship between serum IGF-1 or DHEAS levels and muscle strength or physical performance after adjusting for potential confounders. In multivariate linear regression analysis, there is a significant association between IGF-1, DHEAS and muscle strength using model 1 (adjust for BMI, smoking status, alcohol use, and PA) and model 2 (additionally adjusted for comorbidities) both in males and females. However, results produced using model 3 (additionally adjust for age), were not significant. The similar results were also observed in TUGT and 4-m gait speed using multiple logistic regression analysis. These data suggest that positive relationship between anabolic biomarkers and muscle strength or physical function in older adults may be mediated by age.

The major advantage of our study was using a combination of catabolic and anabolic markers to evaluate the relationship between biomarkers concentration and muscle strength or physical performance. We found that inflammation, growth factor, and androgen signaling factors simultaneously related to aging, and also significantly correlated with one other. In our study, we demonstrated that higher catabolic biomarker levels were related to lower muscle strength, and we did not find that lower anabolic biomarkers (IGF-1 or DHEAS) were related to decreased muscle strength. One explanation for this may be age, which likely contributes to the relationship between anabolic biomarkers and muscle strength or physical function. Another explanation may be that, although we used a well-extablished standard measurement, the handgrip strength test may not be sensitive enough to capture true maximal strength. Moreover, after combining tertiled biomarkers, we did not find any significant associations between biomarker scores and muscle strength or physical performance, suggesting that the impact of anabolic biomarkers on muscle function are trivial and might attenuate the relationship between catabolic biomarkers and muscle strength or physical performance.

Limitations of this study include the fact that it was cross-sectional; therefore, we cannot draw causative conclusions about relationships between muscle strength or physical performance and biomarkers. Moreover, although in this study we demonstrated that there are significant association of inflammation (as indexed by CRP) with muscle strength and physical performance, other inflammation factors such as IL-6 and TNF-a were not measured. Although we adjusted for several confounders, other unmeasured factors might have affected the association between grip strength and inflammatory markers. It is possible that elevated CRP levels reflect sub-clinical disease processes that might be the cause of muscle strength loss [5]. We were also unable to account for severity of disease.

## Conclusion

In conclusion, a higher serum CRP concentration is independently related to lower muscle strength and physical performance in older males. Considering that the muscle strength and physical performance are parts of the components of sarcopenia, and the health consequences of sarcopenia are increasingly being recognized. These results suggest that maintaining lower serum levels of catabolic biomarkers like CRP may contribute to higher muscle strength and improvement of adverse sarcopenia-related outcomes. Additional well-designed clinical studies or prospective interventional studies are necessary to confirm these findings.

## Abbreviations

CRP: C-reactive protein
IGF-1: Insulin-like growth factor-1
DHEAS: Dehydroepiandrosteronesulphate
BMI: Body mass index
TUGT: Timed up and go test
IL-6: Interleukin-6
MET: Metabolic equivalent
PA: Physical activity
IPAQ: International Physical Activity Questionnaire
ANCOVA: Analysis of covariance
SRC: Standard regression coefficient
PKB: Protein kinase B
NFκB: Nuclear factor-κB
